# Time-resolved 3D-CMR using free-breathing 2D-acquisitions

**DOI:** 10.1186/1532-429X-16-S1-P50

**Published:** 2014-01-16

**Authors:** Xiaoguang Lu, Peter Speier, Marie-Pierre Jolly, Hasan Cetingul, Michaela Schmidt, Christoph Guetter, Carmel Hayes, Arne Littmann, Qiu Wang, Mariappan S Nadar, Frank Sauer, Edgar Mueller

**Affiliations:** 1 Imaging and Computer Vision, Siemens Corporate Research, Princeton, New Jersey, USA; 2 MR Application & Workflow Development, Siemens AG, Erlangen, Germany

## Background

A typical CMR exam consists of a limited number of 2D scans that provide standard views of the heart. Diagnosis is limited to these select views. For the acquisition, multiple breath-holds are required - a challenge for many patients. As an improvement, we have investigated a free-breathing (FB) 2D acquisition protocol in conjunction with a novel reconstruction approach. The method provides 3D+time cine data with full heart coverage while simplifying the acquisition.

## Methods

FB images were acquired with a fluoroscopic radial bSSFP, image rate 40-50 ms, res. (1.82 × 1.82 × 7)mm^3^. ECG time-after-trigger was recorded for retrospective mapping to cardiac phases. Volume coverage was achieved by changing the scan plane position or orientation between images slightly in order to not disturb the spin steady state. Images were reconstructed offline with a Compressed Sensing algorithm based on [Liu J et.al., ISMRM, Melbourne, Australia, 2012, P.4249]. Two datasets with approximately orthogonal scan planes were acquired: a short-axis (SX) set covering the heart with steps of 0.05 mm, and a long-axis (LX) set, rotating 180° around the left-ventricular long axis in steps of 0.1°. Our reconstruction pipeline consists of five modules. Modules b)-d) are used to reconstruct a volume at one cardiac phase. The pipeline is described for reconstructing SX data using LX anchors. Modules: a) For each acquired cardiac cycle, the cardiac phase is normalized to a 30 ms time step by deformation-field-assisted interpolation. b) Two approximately orthogonal LX slices, with most consistent SX-LX data correlation at the intersection lines (CAI) over all cardiac phases, are selected as anchors. c) CAI analysis computes the correlation along the intersection line between LX anchors and SX slices, and selects SX candidates with high correlation that actually correspond to SX slices on a similar respiratory phase. d) Residual misalignments between selected SX slices are iteratively corrected by optimizing the correlation with LX anchors while being constrained by neighboring SX slices through non-rigid registration. e) Standard scattered interpolation is applied to interpolate voxel intensities on a regular 1.82^3^mm^3 ^3D grid.

## Results

The proposed automatic pipeline as a prototype was implemented in Matlab [Mathworks, Inc., MA, USA] and successfully applied to four volunteer data sets to generate 3D+time data on a 1.82^3^mm^3 ^grid based on SX data with LX anchors. The average distance between contributing SX slices was ~3.14 mm (see Figure 2).

**Figure 1:**
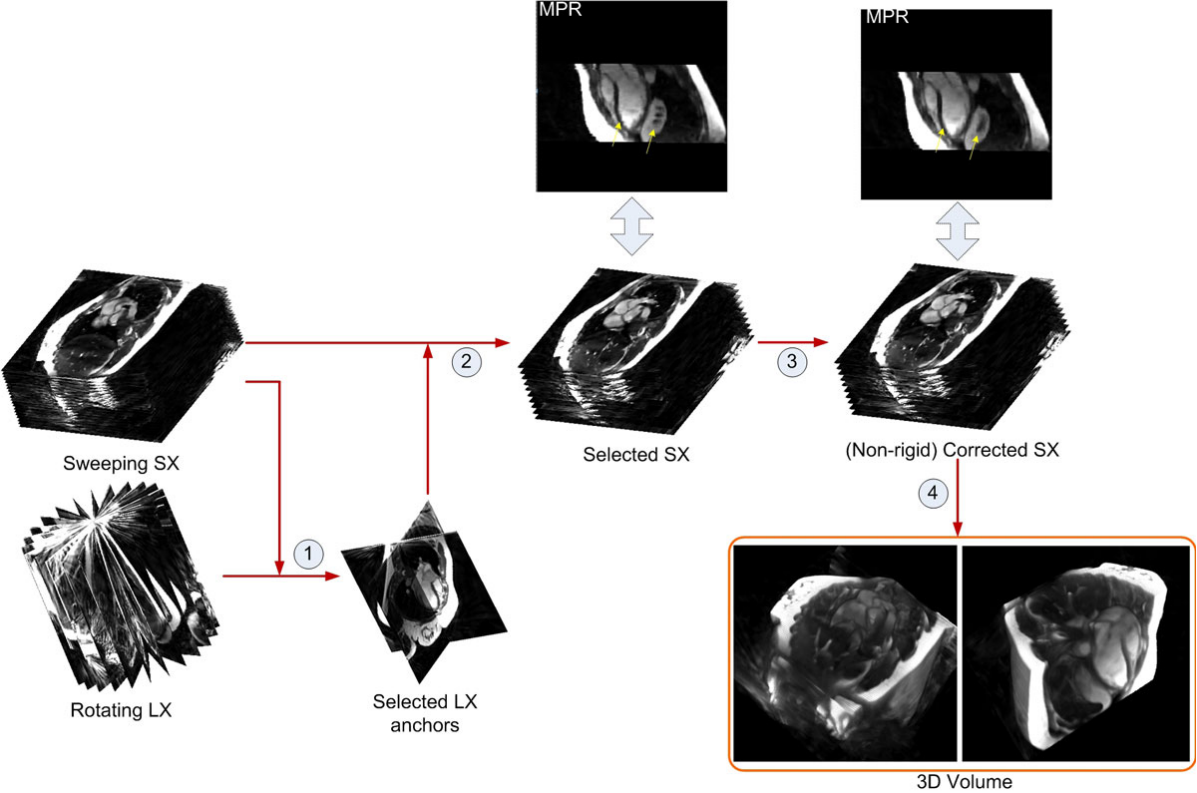
**Volume reconstruction at one cardiac phase with four major steps: 1**. Anchor selection; 2. Slice selection with anchors; 3. Non-rigid slice correction; 4. Scattered interpolation.

**Figure 2:**
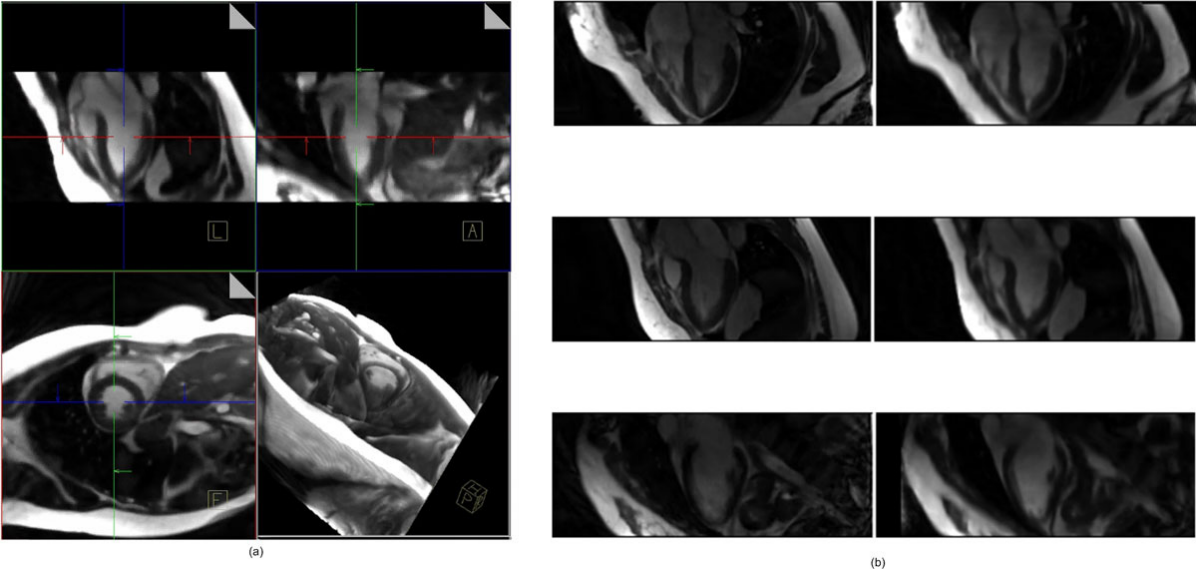
**Example of reconstruction results: a) three orthogonal MPRs and a 3D rendering; b) three MPRs from reconstructed volumes (right) compared with acquired LX images at the same views (left)**.

## Conclusions

We demonstrated the feasibility of fully automated reconstruction of 3D CINEs from orthogonal 2D-FB acquisitions. Further investigations to characterize the performance and robustness of the method are underway.

## Funding

This work is sponsored by Siemens.

